# Persistent Inflammation in the CNS during Chronic EAE Despite Local Absence of IL-17 Production

**DOI:** 10.1155/2013/519627

**Published:** 2013-06-26

**Authors:** Sofia Fernanda Gonçalves Zorzella-Pezavento, Fernanda Chiuso-Minicucci, Thais Graziela Donegá França, Larissa Lumi Watanabe Ishikawa, Larissa Camargo da Rosa, Camila Marques, Maura Rosane Valerio Ikoma, Alexandrina Sartori

**Affiliations:** ^1^Department of Microbiology and Immunology, Biosciences Institute, Universidade Estadual Paulista (UNESP), 18618-070 Botucatu, SP, Brazil; ^2^Laboratório de Citometria de Fluxo-Fundação Dr. Amaral Carvalho, Jaú, SP, Brazil

## Abstract

Experimental autoimmune encephalomyelitis (EAE) is an artificially induced demyelination of the central nervous system (CNS) that resembles multiple sclerosis in its clinical, histopathological, and immunological features. Activated Th1 and Th17 cells are thought to be the main immunological players during EAE development. This study was designed to evaluate peripheral and local contribution of IL-17 to acute and chronic EAE stages. C57BL/6 mice were immunized with MOG plus complete Freund's adjuvant followed by pertussis toxin. Mice presented an initial acute phase characterized by accentuated weight loss and high clinical score, followed by a partial recovery when the animals reached normal body weight and smaller clinical scores. Spleen cells stimulated with MOG produced significantly higher levels of IFN-**γ** during the acute period whereas similar IL-17 levels were produced during both disease stages. CNS-infiltrating cells stimulated with MOG produced similar amounts of IFN-**γ** but, IL-17 was produced only at the acute phase of EAE. The percentage of Foxp3+ Treg cells, at the spleen and CNS, was elevated during both phases. The degree of inflammation was similar at both disease stages. Partial clinical recovery observed during chronic EAE was associated with no IL-17 production and presence of Foxp3+ Treg cells in the CNS.

## 1. Introduction

Multiple sclerosis (MS) is a progressive inflammatory disorder of the central nervous system (CNS) that chronically affects both brain and spinal cord. The pathological hallmark of this pathology is an inflammatory plaque that can be detected by histopathological analysis and, more recently, by using magnetic resonance imaging [1–4]. Findings derived from both patients and animals studies indicated the presence of inflammatory cells and their products raising the general accepted hypothesis that this pathology is mediated by myelin self-reactive T cells [5, 6]. These self-aggressive T cells reach the CNS by crossing the blood-brain barrier (BBB) [7, 8]. At the CNS they destroy the myelin sheet leading to signal conduction slowing or even signal block at the site of inflammation [9]. 

 Experimental autoimmune encephalomyelitis (EAE) is an artificially induced demyelination disease of the CNS that resembles MS in its clinical, histopathological, and immunological features [10]. It is induced in susceptible laboratory animals by immunization with proteins from the CNS, such as myelin proteolipid protein, myelin oligodendrocyte glycoprotein (MOG), or myelin basic protein associated with complete Freund's adjuvant (CFA) [11–13]. This experimental disease is also mediated by myelin-specific T cells, which are initially activated at the peripheral lymphoid organs and then reach the CNS by crossing the permeabilized BBB [7, 14]. Depending upon the mice strain and the immunization strategy, EAE will present different courses, portraying an acute, chronic progressive, or relapsing-remitting kind of disease [9, 15]. The EAE model has indubitably provided a lot of information about the inflammatory processes throughout the progression of the disease. MOG-induced EAE in C57BL/6 mice is amongst the most frequently used mouse models for MS studies [10, 16]. Much of our actual knowledge came from investigations done with EAE in mice. Briefly, activated Th1 and Th17 cells are thought to be the main immunological players during EAE and MS development. Many of their effects would be mediated by IFN-*γ* and IL-17, respectively. They would be initially primed by contact with CNS-specific antigens presented by antigen presenting cells (APCs) in peripheral lymphoid organs. These T cells would then cross the BBB and, by recognizing their cognate antigen presented by CNS resident or immigrating APCs, would initiate a local inflammatory process that would, ultimately, destroy myelin and axons [10, 15]. Which subset of helper T cells is most critical for the pathogenesis of EAE is still a subject of intense controversy. Mice deficient in either ROR*γ*t or T-bet are resistant to EAE induction, supporting the opinion that both Th17 and Th1 cells participate in CNS autoimmune pathologies [17, 18]. Association of techniques that allow purification of brain infiltrating cells and FACS analysis have contributed a lot to elucidate the participation of T cell subsets in EAE development [19, 20]. Collectively the findings obtained with these methodologies demonstrated that both autoreactive Th1 and Th17 cells, their balance at the site of inflammation, and their cytokines and chemokines are responsible for CNS autoimmunity. Studies analyzing the phenotype of T cells infiltrating the CNS during EAE revealed the presence of both Th1 and Th17 cells [21, 22]. However, there was an interesting difference in their proportions when distinct mice lines were compared. For example, C57BL/6 mice showed Th1 predominance whereas SJL mice displayed more Th17 in the inflamed CNS during higher clinical scores [21, 22]. The immunization procedure also significantly affected this differential contribution of Th1 and Th17 cells. Mice immunization with similar but distinct MOG epitopes evoked T cell responses characterized by different Th1/Th17 proportions, depending on the avidity of T cells for their corresponding epitopes [23]. It has been hypothesized that this differential ratio of Th1 and Th17 cells in CNS-infiltrating cells could explain the variety of clinical disease manifestations found in MS patients [24]. In this context, the present study was designed to evaluate the peripheral and local contribution of IL-17 to acute and chronic EAE stages in C57BL/6 mice.

## 2. Material and Methods

### 2.1. Animals

Female C57BL/6 mice (8–10 weeks old) were purchased from CEMIB (UNICAMP, São Paulo, SP, Brazil). The animals were fed with sterilized food and water *ad libitum *and were manipulated in accordance with the ethical guidelines adopted by the Brazilian College of Animal Experimentation. All experimental protocols were approved by the local Ethics Committee (Ethics Committee for Animal Experimentation, Medical School, Univ. Estadual Paulista).

### 2.2. EAE Induction

MOG35–55 peptide (MEVGWYRSPFSRVVHLYRNGK) was synthesized by Proteimax, São Paulo, Brazil. EAE was induced as previously described [20]. Briefly, mice were immunized subcutaneously with 150 *μ*g of MOG35–55 peptide emulsified in CFA containing 5 mg/mL of mycobacteria. Mice also received 2 doses, 0 and 48 h after immunization, of 200 ng of *Bordetella pertussis* toxin (Sigma) intraperitoneally. Clinical assessment of EAE was daily performed according to the following criteria: 0—no disease, 1—limp tail, 2—weak/partially paralyzed hind legs, 3—completely paralyzed hind legs, 4—complete hind and partial front leg paralysis, and 5—complete paralysis/death.

### 2.3. CNS-Infiltrating Cells Isolation

Mice were anesthetized with ketamine/xylazine and perfused with 10 mL of saline solution. Brain and cervical spinal cords were excised, macerated, and maintained in 4 mL of RPMI (Sigma) supplemented with 2.5% collagenase D (Roche) at 37°C, 5% CO_2_ incubator. Forty-five min later suspensions were washed in RPMI and centrifuged at 450 ×g for 15 min at 4°C. Cells were resuspended in percoll (GE Healthcare) 37% and gently laid over percoll 70% in tubes of 15 mL. The tubes were centrifuged at 950 ×g for 20 min with centrifuge breaks turned off. After centrifugation the ring containing mononuclear cells was collected, washed in RPMI, and centrifuged at 450 ×g for 5 min. Cellular suspensions were then resuspended in complete RPMI medium, counted, and analyzed.

### 2.4. Cell Culture Conditions and Cytokine Quantification

Control and EAE mice were euthanized 19 days (acute phase) or 30 days (chronic phase) after EAE induction. Lymph nodes (popliteal + inguinal), spleen and CNS-isolated cells were collected and adjusted to 2.5 × 10^6^ cells/mL, 5 × 10^6^ cells/mL, and 2.5 × 10^5^ cells/mL, respectively. Cells were cultured in complete RPMI medium (RPMI supplemented with 5% of fetal calf serum, 20 mM glutamine, and 40 IU/mL of gentamicin). Spleen and lymph node cells were stimulated with MOG (20 *μ*g/mL) and Concanavalin A, Sigma Aldrich (10 *μ*g/mL). CNS-isolated cells were restimulated *in vitro* with 50 *μ*g/mL of MOG. Cytokine levels were evaluated 48 h later by enzyme-linked immunosorbent assay (ELISA) in culture supernatants using IFN-*γ* and IL-10 BD OptEIA Sets (Becton Dickinson) and IL-6, IL-17, and TNF-*α* Duosets (R&D Systems, Minneapolis, MN, USA). The assays were performed according to the manufacturer's instruction. 

### 2.5. Proportion of CD4+CD25+Foxp3+ T Cells

Spleen cells were collected and the red blood cells were lysed with Hank's buffer containing NH_4_Cl. Cells from spleen and cervical spinal cord were obtained as described before and adjusted to 2.5 × 10^6^ cells/100 *μ*L. Spleen and CNS-infiltrating cells were then incubated with 0.5 *μ*g of fluorescein isothyocianate (FITC) anti-mouse CD4 (clone GK1.5) and 0.25 *μ*g of allophycocyanin (APC) anti-mouse CD25 (clone PC61.5) for 20 min at room temperature. A staining for Foxp3 was then performed utilizing the phycoerythrin (PE) anti-mouse/rat Foxp3 Staining Set (eBioscience, San Diego, CA, USA) according to the manufacturer's instructions. After incubation, the cells were fixed in paraformaldehyde 1%. The cells were analyzed by flow cytometry using the FACSCalibur (Becton Dickinson, San Jose, CA, USA) and BD CellQuest Pro software (Becton Dickinson, San Jose, CA, USA). 

### 2.6. Evaluation of Inflammatory Infiltrates in the CNS

A histological analysis was performed in the CNS at the 30th day after EAE induction. After euthanasia and blood withdrawal, brain and lumbar spinal cord samples were removed and fixed in 10% formaldehyde. Tissues were dehydrated in graded ethanol and embedded in a 100% paraffin block. Serial sections with 5 *μ*m thickness were cut and stained with hematoxylin and eosin. Five to six photos were obtained by each animal with a Nikon microscope.

### 2.7. Statistical Analysis

Data were expressed as mean ± SE. Comparisons between groups were made by Student's *t* test or one way ANOVA with post hoc Holm-Sidak test for parameters with normal distribution and by Mann-Whitney *U* test or Kruskal-Wallis test for parameters with non-normal distribution. Significance level was *P* < 0.05. Statistical analysis was accomplished with SigmaStat for Windows v 3.5 (Systat Software Inc). 

## 3. Results

### 3.1. EAE Progression

**Figure 1:**
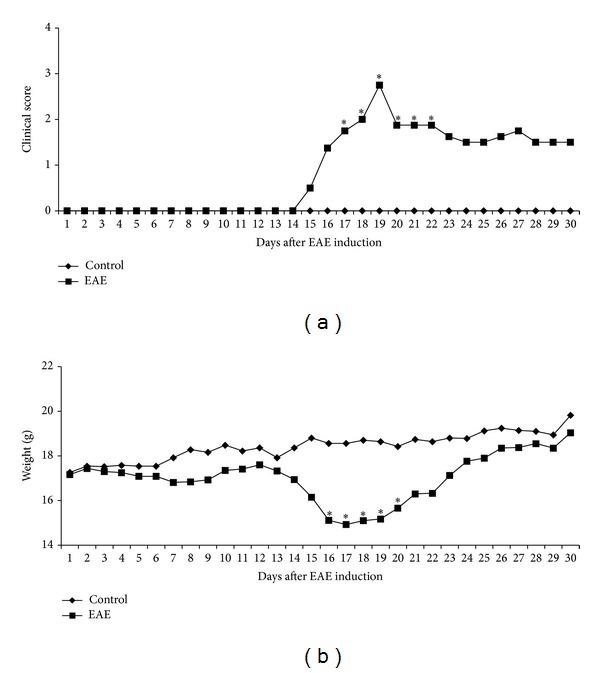
Female C57BL/6 mice were submitted to EAE induction by immunization with MOG emulsified in CFA. Clinical score (a) and weight variation (b) were daily evaluated during 30 days. Data were presented by mean ± SE of 6 mice and representative of two independent experiments. *P < 0.05.

MOG-immunized C57BL/6 mice developed the first signs of EAE around 15 days after immunization by displaying loss of tail tonus. The maximal clinical symptomatology, that is indicative of the acute phase, occurred at day 19 when the average clinical score reached 2.8 (Figure 1(a)). From this period on the animals slightly improved their mobility but did not completely recover from paralysis. The clinical scores that declined to an average of 1.5 did not significantly change until the 30th day that was chosen as the end point of the experiment. Variation in body weight showed an expected course characterized by a significant weight drop during the acute phase (Figure 1(b)). This loss was followed by a progressive weight recovery. Animals with EAE reached weight values similar to the normal control group at the 30th day following immunization. 

### 3.2. Histological Analysis of Brain and Spinal Cord

**Figure 2:**
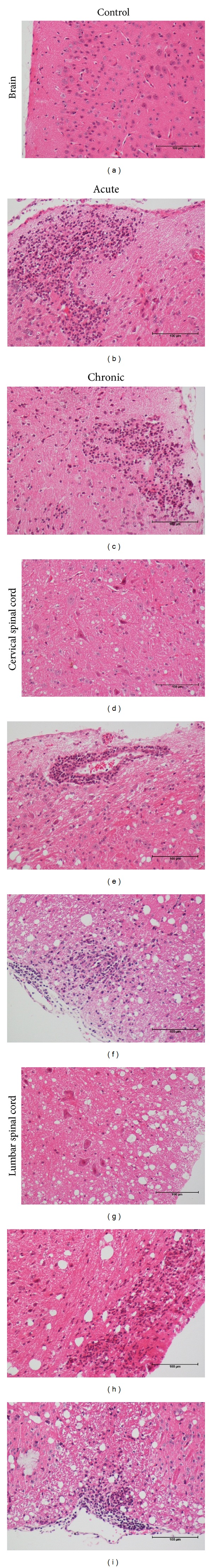
Histopathological analysis of the CNS in C57BL/6 mice with EAE. Female mice were submitted to EAE induction by immunization with MOG emulsified in CFA. Inflammatory infiltrates were evaluated in brain (a, b, and c), cervical (d, e, and f), and lumbar (g, h, and i) spinal cord sections stained with H&E in control animals (a, d, and g) and in animals with EAE during acute (b, e, and h) and chronic disease stages (c, f, and i). Panel is representative of 6 animals/group.

The histological analysis was performed in samples obtained during the acute and chronic phases. Typical lesions, characterized by an intense perivascular inflammatory infiltrate were observed in the brain (Figures 2(a), 2(b) and 2(c)) and also in both, cervical (Figures 2(d), 2(e) and 2(f)) and lumbar sections (Figures 2(g), 2(h) and 2(i)) of the spinal cord. A visual inspection indicated that the degree of inflammation was equivalent in these two clinical disease phases.

### 3.3. Production of Cytokines by Peripheral Lymphoid Organs

**Figure 3:**
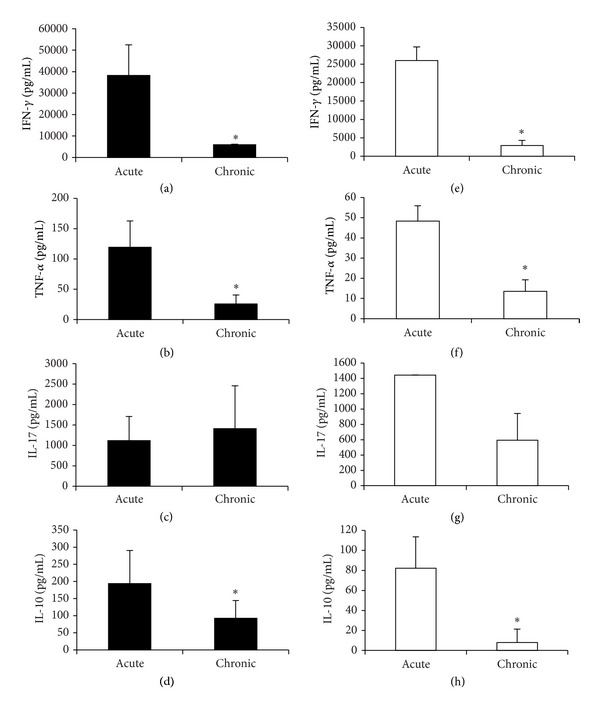
Production of cytokines by peripheral lymphoid organs. C57BL/6 mice were submitted to EAE induction and cytokine production was assayed in acute and chronic EAE stages. IFN-*γ* (a and e), TNF-*α* (b and f), IL-17 (c and g), and IL-10 (d and h) levels were measured in spleen (a, b, c, and d) and lymph node cell (e, f, g, and h) cultures stimulated with MOG. Data were presented by mean ± SE of 6 mice and representative of two independent experiments. *P < 0.05.

Cytokine production by peripheral lymphoid organs was compared during acute and chronic phases of the disease. The profile of cytokine production induced by MOG was very similar in spleen and lymph node cell cultures. Elevated levels of IFN-*γ* (Figures 3(a) and 3(e)), TNF-*α* (Figures 3(b) and 3(f)), and IL-10 (Figures 3(d) and 3(h)) were present in both phases; however, their values were significantly higher in cultures from acute phase animals. IL-17 (Figures 3(c) and 3(g)) was also elevated during the acute phase but, differently from the other cytokines, its levels remained elevated during the chronic period, presenting no statistical difference in comparison to the acute phase of the EAE.

### 3.4. Production of Cytokines by CNS Infiltrating Cells

**Figure 4:**
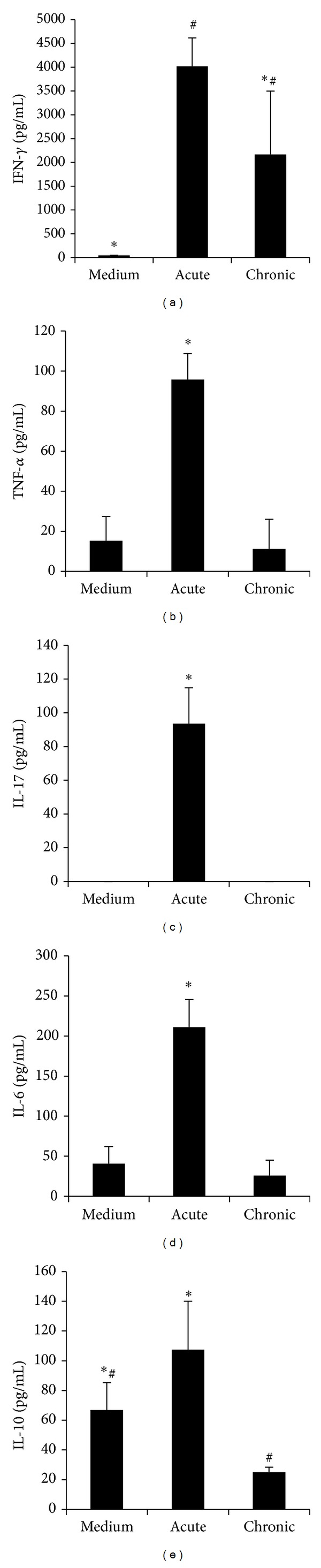
Production of cytokines by CNS infiltrating cells. Female C57BL/6 mice were submitted to EAE induction by immunization with MOG emulsified in CFA and the cytokine production was assayed in acute and chronic stages of EAE. IFN-*γ* (a), TNF-*α* (b), IL-17 (c), IL-6 (d), and IL-10 (e) production by cells eluted from the brain stimulated *in vitro* with MOG. Data were presented by mean ± SE of 6 mice and representative of two independent experiments. * and ^#^ *P* < 0.05.

Cytokine production by cells eluted from the CNS stimulated with MOG presented a different behavior, depending upon the cytokine that was being analyzed. TNF-*α* (Figure 4(b)) and IL-6 (Figure 4(d)) were significantly higher during the acute phase in comparison to the chronic period of the disease. Detectable levels of these cytokines were also present in nonstimulated cultures. IL-10 production showed a similar profile with significantly higher production during the acute phase; however, in this case the spontaneous production of this cytokine was very high approaching the levels found in MOG stimulated cultures (Figure 4(e)). IFN-*γ* levels were, differently from the other cytokines, similarly elevated in both phases with no spontaneous release in culture (Figure 4(a)). IL-17 production presented a completely distinct profile characterized by significantly elevated levels at the acute disease and no production at the chronic phase (Figure 4(c)). 

### 3.5. Quantification of Foxp3+ T Cells

**Figure 5:**
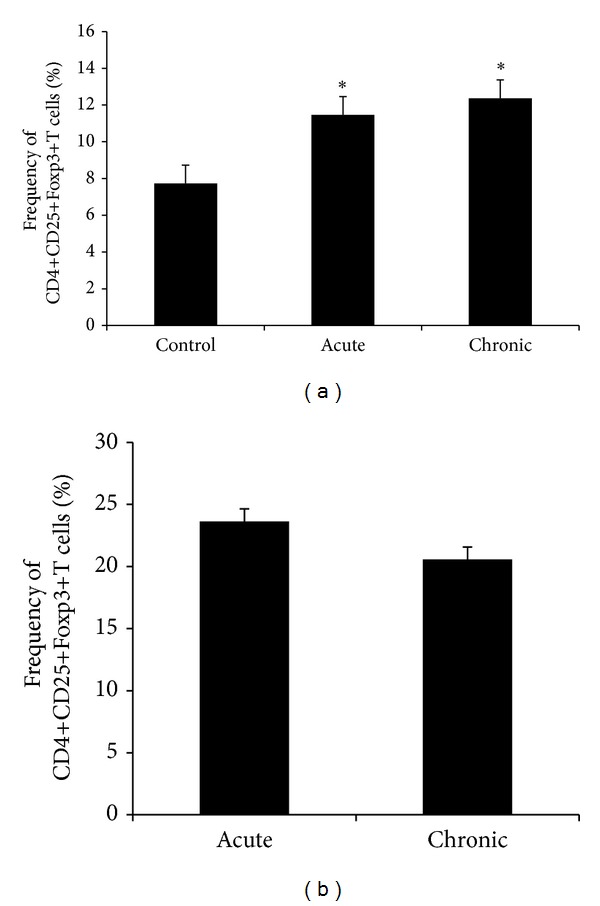
Frequency of CD4+CD25+Foxp3+ T cells in spleen (a) and in CNS (b). The percentage of CD4+CD25+Foxp3+ T cells was determined during acute and chronic stages of EAE by flow cytometric analysis. Data were presented by mean ± SE of 6 mice and representative of two independent experiments. *P < 0.05.

The frequency of CD4+CD25+Foxp3+ T cells was investigated at the spleen and also at the mononuclear cells eluted from the CNS tissue. As can be observed in Figure 5(a), there was a small but significant increase in the Foxp3+ T cell subset, in the spleen, during both phases of EAE development in comparison to normal animals. The analysis made in the CNS cells also revealed the presence of this regulatory T cell subset in both phases (Figure 5(b)). However, differently from the findings in the periphery, there was a drop in Foxp3+ T cells during the chronic EAE phase.

## 4. Discussion

The main goal of this study was to compare the contribution of IL-17 and IFN-*γ* to inflammation observed during acute and chronic EAE. The relevance of this investigation resides in the fact that IL-17 is a relatively recent described cytokine whose role in autoimmune pathologies, including EAE, is not entirely known [10]. In addition, it is highly possible that the relative proportion of IL-17 and IFN-*γ* at the CNS is associated with the variety of the human disease clinical manifestations [25].

 In preliminary assays we established that immunization of female C57BL/6 mice with MOG associated with CFA triggered a classical EAE disease, characterized by ascending paralysis. Two very distinct disease stages were observed: an acute phase and a chronic phase. The acute phase was characterized by the highest clinical scores and the most significant weight loss. The disease peak (around the 19th day) was followed by a slow drop in clinical score that stabilized around the 30 day after immunization. This second period that was characterized by lower clinical scores and a complete body weight recovery was understood as the chronic EAE stage. A plethora of experimental models is being explored to unravel the immunopathogenetic mechanisms responsible for MS beginning and progression [26]. The model used by us allowed a clear differentiation between acute and chronic phases as has already been described [27]. On the other hand, other authors described a different profile, with no distinction between these two phases [28].

 All the following approaches were done with the purpose to compare these two EAE stages. The clinically most severe acute period was concomitant with a higher production of IFN-*γ*, TNF-*α*, and IL-10 by peripheral lymphoid organs, compared to the chronic phase. Interestingly and differently from these cytokines, the production of IL-17 remained as elevated in the chronic phase as it was during the acute period. This differential production of IFN-*γ* and IL-17 during chronic EAE could be explored as a marker to follow MS evolution and, maybe, as an indicator for treatment efficacy. The lower production of some cytokines during the chronic period could result from induction of cells able to regulate the immune response. In accordance with this possibility, more elevated levels of regulatory Foxp3+ T cells were found in the spleen of diseased animals, in both phases, in comparison to normal control mice. Induction, expansion, and maintenance of a putative population of Treg cells have been intensely investigated in EAE [29]. However, this seems to be the first report comparing Foxp3 T cell levels during these two disease phases.

 Unexpectedly, the lower clinical scores and decreased cytokine production in the periphery during the chronic period were not associated with a significant decrease in the CNS inflammatory reaction. A visual analysis of the inflammatory infiltrates at both brain and spinal cord (cervical and lumbar) did not reveal a great difference in the degree of cellular infiltration. The most immediate explanation for these findings would be a qualitative difference in these inflammatory infiltrates, rather than the expected downregulation of inflammation during the chronic phase. To confirm this hypothesis we evaluated cytokine production by cells infiltrated in the brain tissue. Production of cytokines by cells isolated from the CNS has greatly contributed to clarify the participation of these molecules in EAE and MS pathologies. This technique allows the most direct and desirable investigation of what happens *in situ*, in the focus of inflammation and demyelination [30]. The local production of some cytokines presented a pattern that resembled the one produced by peripheral cells. TNF-*α*, IL-6, and IL-10 were found in much higher levels during the acute phase. However, IFN-*γ* and IL-17 presented very distinct production patterns, in comparison to their production by peripheral cells. IFN-*γ* was produced in significant amounts in both stages whereas IL-17 was released only during the acute phase. Considering the local cytokine production, the most intriguing finding of our work was this apparent local disappearance of IL-17 producer cells during the chronic phase of the disease. This was considered a relevant finding because it has many implications in the actual status of knowledge in this area. Similar findings were reported by other authors. In acute EAE, a high number of CNS autoreactive Th17 cells are present in the inflamed CNS. High levels of CNS autoreactive Th17 cells are still present in the immune periphery but not in the CNS during EAE recovery period [28, 31]. This finding is also similar to reports made with CNS samples from MS patients. IL-17 and IFN-*γ* production by T cells has been associated with disease activity in MS patients and is also expressed in brain lesions. In addition, IL-17 expression in MS brain lesions [32, 33] and enrichment of IL-17-producing cells in glial cells, CD4+ and CD8+ T cells, were demonstrated by microarray analysis in the active rather than inactive areas of MS brain lesions [34]. Elevated frequencies of IL-17-producing cells have been associated with disease activity in the peripheral blood of MS patients [35, 36]. Interestingly, it has also been reported that although IL-17 and IFN-*γ* were elevated early during the disease, only IFN-*γ* enhancement was associated with relapse [37].

 To try to understand the reduced local production of cytokines we checked the presence of T cells with presumed regulatory activity. Indeed, CD4+CD25+Foxp3+ T cells were found in the CNS, in both phases of the disease. Around 31% and 28% of the cells infiltrated in the brain, during the acute and chronic phases, respectively, were Foxp3+ regulatory T cells. These results are consistent with previous reports demonstrating the pivotal role of these cells in EAE control. It has been demonstrated that myelin-specific Treg cells are able to migrate and to accumulate in the CNS in animals with EAE [22, 27, 38]. In addition, higher frequency of Treg cells in the CNS have consistently been shown to correlate with recovery from EAE [22, 27]. These cells were not, however, sufficient to completely control the function of encephalitogenic T effector cells since the acute phase was followed by a chronic phase characterized by partial paralysis and persistent inflammation. These findings were very similar to the ones described by Korn et al., 2007 [22]. These authors clearly demonstrated that the expansion of Foxp3+ cells in the periphery was followed by their accumulation in the CNS. They also suggested that the inflammatory microenvironment was probably hindering the effective control of the autoimmune reaction by Foxp3+ cells.

Interestingly, this chronic inflammatory infiltrate was clearly distinct from the one observed during the acute disease phase. In this case, as described previously, there was still a local production of IFN-*γ* but not of IL-17. The mechanism of this change was not investigated. However, previous reports suggest that myeloid cells and the well-known plasticity of Th17 cells could be involved in this phenomenon. Myeloid effector populations present in the CNS include resident activated microglia and blood-derived monocytes, macrophages, and DCs [39]. These cells clearly mediate destruction of myelin sheets and axons in both MS and EAE [40]. However, convincing data indicate that they play a dual role. They initially promote T cell function by acting as APCs and also as effector cells activated by T lymphocytes. On the other hand, highly activated T cells trigger activation, including NO synthesis, of these myeloid cells which, in turn, suppress ongoing T cell activity [41]. Myeloid cells as macrophages and DCs have been both involved in this contraction of the local immune response in EAE. Recently, in the EAE rat model, it was demonstrated that classically activated macrophages (M1) play a major pathogenetic role in disease initiation whereas alternatively activated macrophages (M2) contribute to disease recovery [42]. Procedures targeting the shift from M1 to M2 macrophages clearly reduced EAE severity in mice [43].

 The contribution of myeloid-derived suppressor cells (MDSCs) to control EAE during the chronic phase is also supported by recent data. Moliné-Velázquez et al., 2011 [44], described that MDSCs limit neuroinflammation by promoting apoptosis of T lymphocytes in the spinal cord of mice with EAE. Ioannou et al., 2012 [45], demonstrated that granulocytic MDSCs accumulate within the CNS before EAE remission. Even more convincing was their observation that transfer of these cells was able to determine clinical improvement, decreased demyelination, and also inhibition of encephalytogenic Th1 and Th17 types of response.

Regarding Th17 plasticity, human and murine Th17 committed lymphocytes can turn to Th1 cells by upregulating T-bet and IFN-*γ* and downregulating IL-17 in the presence of Th1 polarizing factors [46, 47]. This *in vivo* plasticity was clearly demonstrated by Hirota et al, 2011 [48], by using an IL-17A reporter mouse. These authors showed that up to two-thirds of CNS-infiltrating Th17 cells, in mice with MOG-induced EAE, turned to Th1 cells by expressing their signature cytokine (IFN-*γ*). In this scenario we could think that the plasticity of Th17 cells is another factor that contributes to the observed absence of IL-17 during the chronic EAE phase.

Administration of IL-17 or adoptive transfer of Th17 myelin specific cells, during this chronic EAE stage, could certainly shed some light on this complex interplay of T cell subsets. This kind of analysis was not yet accomplished by us or other research groups. Even though this is a very unpredictable and complex subject, we are initially inclined to believe that both procedures would trigger similar outcomes; that is, they would exacerbate the disease. This prognosis is based on the literature data that shows improved clinical conditions upon IL-17 neutralization [28] and disease exacerbation after adoptive transfer of myelin-specific Th17 cells [49]. However, it is important to have in mind that Th17 transfer would include the contribution of the other cytokines produced by this Th subset [50]. In addition, Th17 cells would meet an inflammatory microenvironment in the brain that could affect their activity.

## 5. Conclusions

Together, these results demonstrated that chronic EAE phase, characterized by evident inflammatory infiltrates in the brain, is associated with persistent and high local IFN-*γ* production, absence of IL-17 synthesis, and local permanency of a high percentage of T CD4+CD25+Foxp3+ regulatory cells.
